# Supplementary material for: Widespread natural methane and oil leakage from sub-marine Arctic reservoirs
Source: Nat Commun. 2023 Mar 30;14:1782. doi: 10.1038/s41467-023-37514-9 (PMC10063646; doi:10.1038/s41467-023-37514-9)
Supplement: Supplementary file 1 — Supplementary Information [file 41467_2023_37514_MOESM1_ESM.pdf]

Supplementary Information

Widespread natural methane and oil leakage from sub-marine Arctic reservoirs

Pavel Serov*[1], Rune Mattingsdal[2], Monica Winsborrow[1], Henry Patton[1], Karin Andreassen[1]

1 CAGE - Centre for Arctic Gas Hydrate, Environment and Climate, UiT – The Arctic University of Norway, Tromsø, Norway

2 NPD – Norwegian Petroleum Directorate, Harstad Office, Harstad, Norway

* Corresponding Author. Pavel Serov. pavel.russerov@uit.no

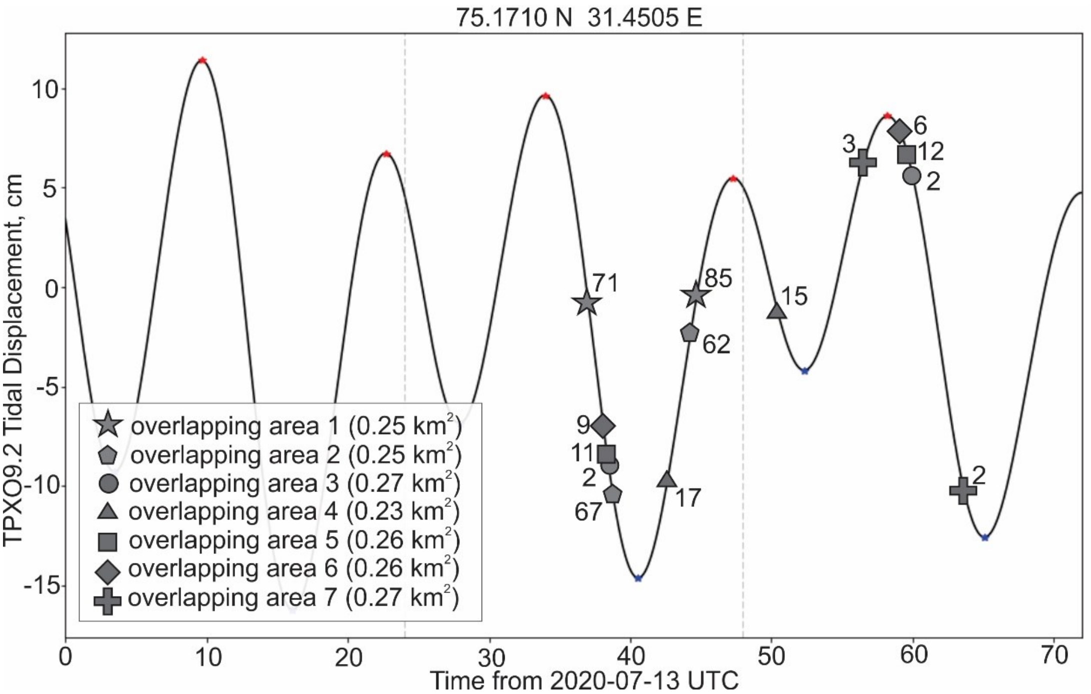

Fig. S1: number of gas flares identified during echosounder surveys repeated at variable tidal displacement. Tidal displacement data is from TPXO9.2[1]. Source data are provided as a Source Data file.

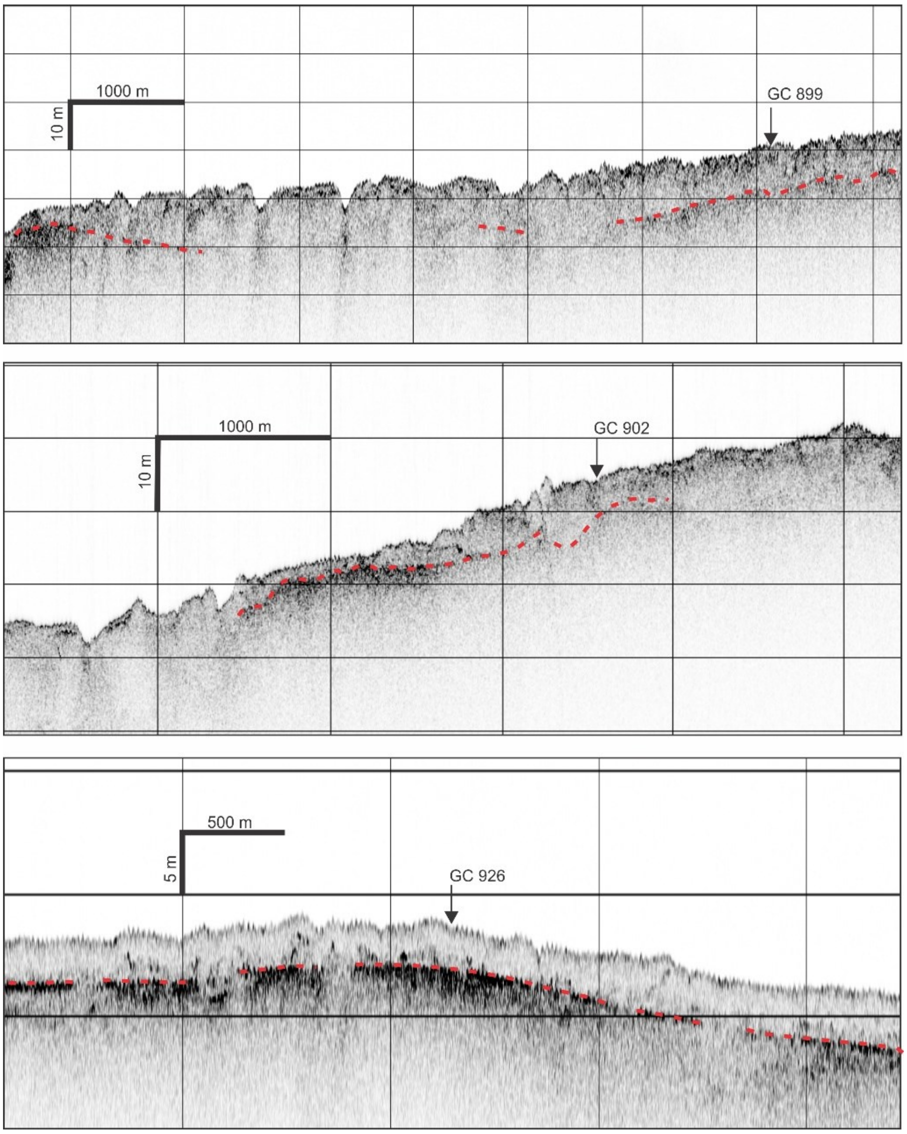

Fig. S2: Fragments of Chirp profiles across sediment sampling locations. Dashed red line highlighs acoustically hard boundary between glacigenic deposits and underlying hard substrate of sediemntary rocks.

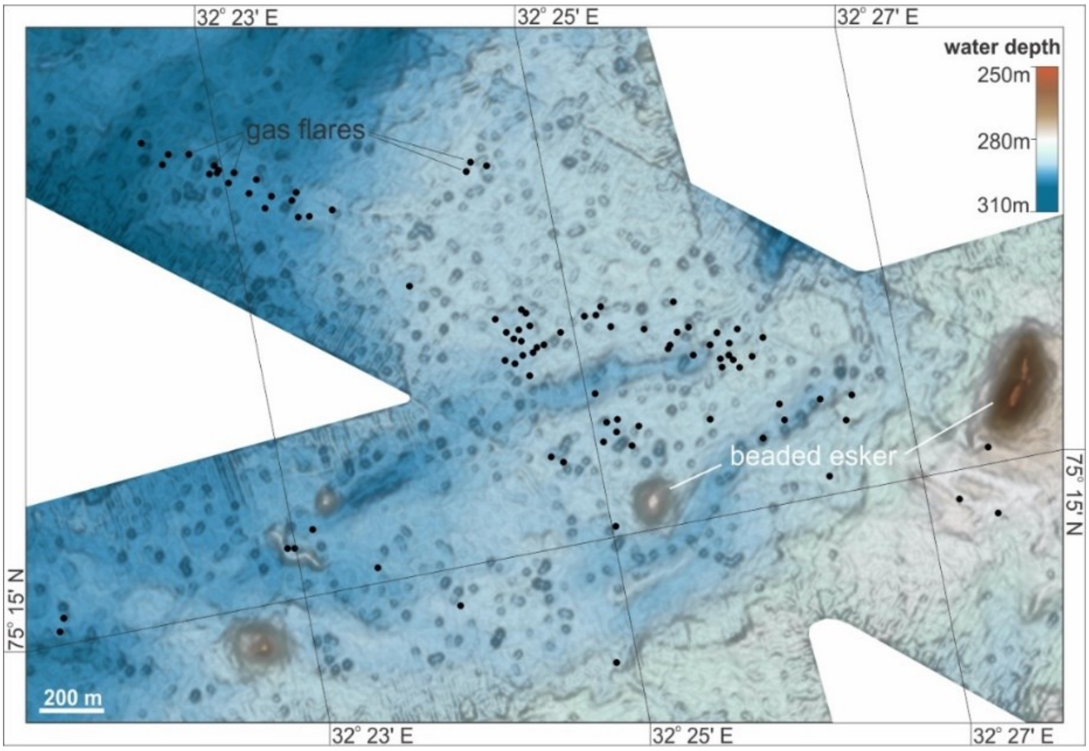

Fig. S3: Field of pockmarks on seabed topography (3 m horizontal resolution) and location of gas flares.

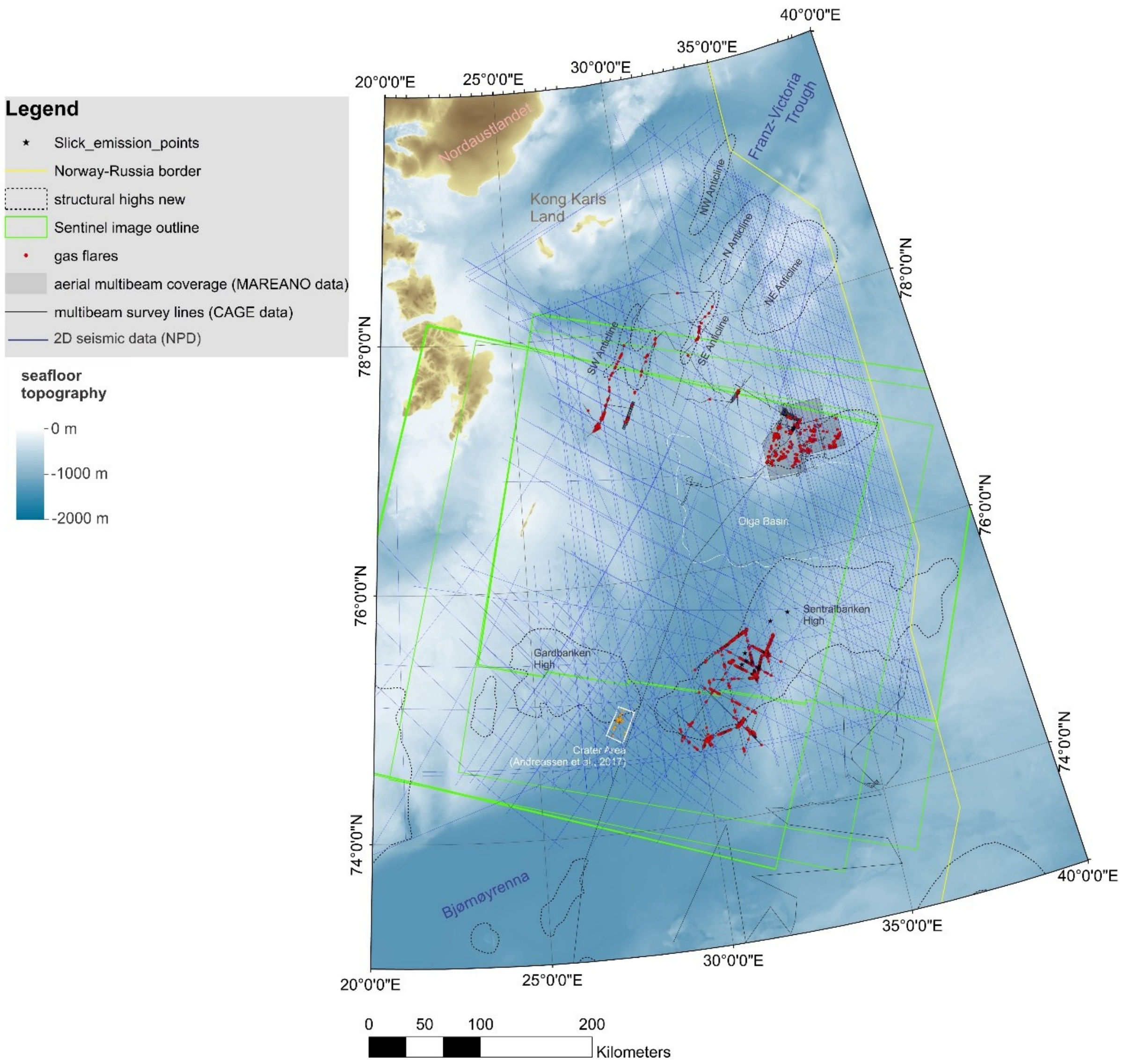

Fig. S4: Data coverage. Topographic data is from IBCAO V4[2]

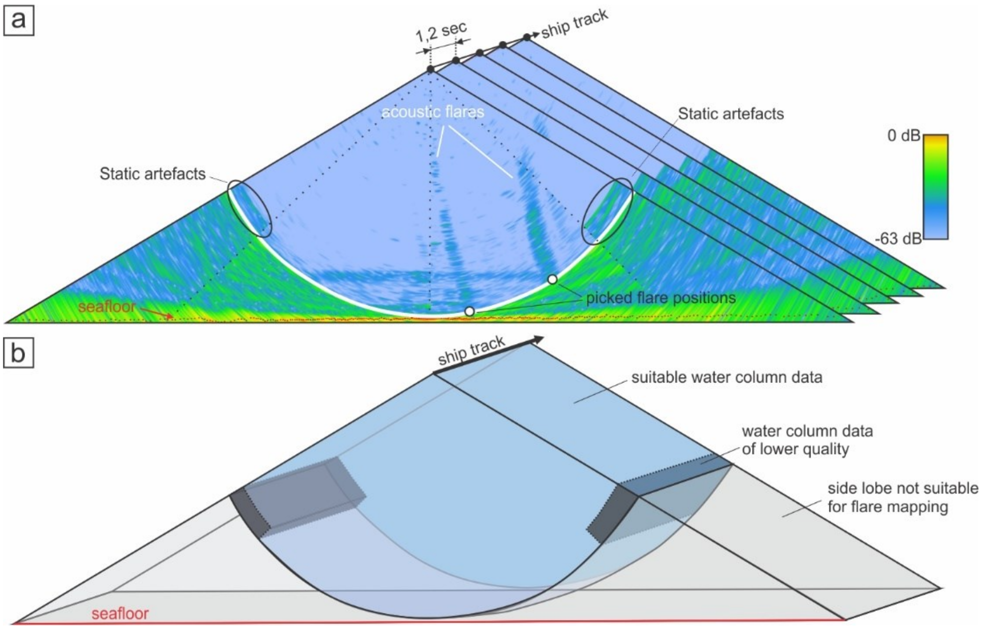

Fig. S5: Water column data acquired with Kongsberg EM302 multibeam system at ~302 m water depth. a Consequential water column images showing advantages (broad data coverage close to the seafloor, high sensitivity to gas in water) and limitations (static artifacts, side-lobe artifacts) of the data. Solid white line shows minimum slant range limits; radiating black dashed lines separate 4 sectors of the transmit fan. b Volume of the water column data suitable for acoustic flare mapping.

Supplementary Reference:

1 Egbert, G. D. & Erofeeva, S. Y. Efficient Inverse Modeling of Barotropic Ocean Tides. *Journal of Atmospheric and Oceanic Technology* **19**, 183-204 (2002). https://doi.org:10.1175/1520-0426(2002)019<0183:EIMOBO>2.0.CO;2

2 Jakobsson, M. *et al.* The International Bathymetric Chart of the Arctic Ocean Version 4.0. *Scientific Data* **7**, 176 (2020). https://doi.org:10.1038/s41597-020-0520-9
